# The RTR Complex Partner RMI2 and the DNA Helicase RTEL1 Are Both Independently Involved in Preserving the Stability of 45S rDNA Repeats in *Arabidopsis thaliana*

**DOI:** 10.1371/journal.pgen.1006394

**Published:** 2016-10-19

**Authors:** Sarah Röhrig, Susan Schröpfer, Alexander Knoll, Holger Puchta

**Affiliations:** Botanical Institute II, Karlsruhe Institute of Technology, Karlsruhe, Germany; Weizmann Institute, UNITED STATES

## Abstract

The stability of repetitive sequences in complex eukaryotic genomes is safeguarded by factors suppressing homologues recombination. Prominent in this is the role of the RTR complex. In plants, it consists of the RecQ helicase RECQ4A, the topoisomerase TOP3α and RMI1. Like mammals, but not yeast, plants harbor an additional complex partner, RMI2. Here, we demonstrate that, in *Arabidopsis thalian*a, RMI2 is involved in the repair of aberrant replication intermediates in root meristems as well as in intrastrand crosslink repair. In both instances, RMI2 is involved independently of the DNA helicase RTEL1. Surprisingly, simultaneous loss of RMI2 and RTEL1 leads to loss of male fertility. As both the RTR complex and RTEL1 are involved in suppression of homologous recombination (HR), we tested the efficiency of HR in the double mutant *rmi2-2 rtel1-1* and found a synergistic enhancement (80-fold). Searching for natural target sequences we found that RTEL1 is required for stabilizing 45S rDNA repeats. In the double mutant with *rmi2-2* the number of 45S rDNA repeats is further decreased sustaining independent roles of both factors in this process. Thus, loss of suppression of HR does not only lead to a destabilization of rDNA repeats but might be especially deleterious for tissues undergoing multiple cell divisions such as the male germline.

## Author Summary

The Bloom syndrome and Hoyeraal Hreidarsson syndrome are severe diseases in humans that are correlated with genome instability. Interestingly, plants harbor homologs of factors that are defective in the respective diseases. In the model plant *A*. *thaliana* these proteins play important roles in various aspects of the repair of genetic information and the maintenance of repetitive elements. Here, we show that the concomitant loss of function of two specific factors that are representative for each syndrome leads in plants to male sterility, due to somatic catastrophe leading to instability and cell death. This defect is correlated with a massive loss of repetitive genes involved in general protein production. It has been shown before for mammals that loss of certain other factors involved in genome stability leads to a defect in neural development. Our results now demonstrate that genome instability can also result in organ-specific defects in plants, in our case during flower development, leading to a defect in the cell proliferation of the premeiotic male germline.

## Introduction

Genomic stability is thoroughly maintained by repair mechanisms such as HR to process pervasive DNA lesions in somatic cells. Different HR sub-pathways can process double-strand breaks and blocked replication forks while forming recombination intermediates, e.g., displacement loops (D-loops) and double Holliday Junctions (dHJs). One HR mechanism to process recombination intermediates, namely the dissolution pathway, is mediated by the RTR complex and resolves branched DNA structures exclusively without reciprocal exchange between chromosome arms to non-crossover products. The complex is also involved, via its helicase function, in the regulation of D-loop formation [1].

The corresponding mammalian BTR complex consists of the Bloom syndrome helicase BLM, the type IA topoisomerase TOP3α and two OB-fold containing structural proteins RMI1 and RMI2. BLM mediates branch migration to form a hemicatenane structure during dissolution of dHJs [2,3]. The hemicatenane can then be processed through decatenase activity of the TOP3α, supported by RMI1 and RMI2 [4–8]. Both of the structural proteins RMI1 and RMI2 have been shown *in vitro* to form a sub-complex without catalytic function itself but that is essential for proper dissolution [9,10]. Characterization and identification of RMI2 in human cell culture revealed a supportive role for RMI2 in complex assembly and function [7,8]. Inactivation of RMI2 in chicken DT40 cells results in an increased level of sister chromatid exchange (SCE), which is characteristic of Bloom syndrome cells. RMI2 is an integral component of the BTR complex, as demonstrated by immunoprecipitation analysis [8].

Homologues of all mammalian complex partners were identified for *A*. *thaliana*. The RecQ helicase AtRECQ4A is the functional counterpart of BLM in plants, and respective T-DNA insertion mutants revealed hypersensitivity against DNA-damaging agents and elevated frequency of HR in somatic cells [11–13]. Likewise, loss of the homologous complex partners AtTOP3α and AtRMI1 causes hyper-recombination and hypersensitivity against DNA-damaging agents. In addition to their roles in somatic cells, both proteins, but not AtRECQ4A, are essential for meiotic recombination in plants. A defect in either protein leads to severe chromosomal fragmentation resulting in arrest at the end of meiosis I and sterility [14–16]. Recent results in yeast indicate that Top3 and Rmi1, but not Sgs1 (the BLM homologue of yeast), also function in the dissolution of late meiotic recombination intermediates [17–19]. A plant homologue of RMI2 was identified by a homology search [8] and proteomics analysis [20], but its functional role has not yet been analyzed. Therefore, it is important to define the role of RMI2 in *A*. *thaliana* in somatic DNA repair as well as in meiosis.

Apart from the RTR complex, resolution of recombination and repair intermediates can be mediated by different types of nucleases. The MUS81-EME1 complex can process branched DNA structures by dual incisions that generate either crossover (CO) or non-crossover (NCO) products [21–23]. A defect in MUS81-EME1 leads to hypersensitivity against methyl methane sulfonate (MMS) in respective cells, indicating an important role in maintaining aberrant replication forks. In *Arabidopsis*, the MUS81 homologue is involved in the repair of different types of DNA lesions that are induced by MMS and the crosslinking agent *cis*-platin (*cis*-Pt) [24]. A defect in both repair mechanisms leads to synthetic lethality, as observed in the double mutant *recq4A-4 mus81-1*. This indicates that both proteins work in parallel pathways to resolve recombination intermediates [24].

In *A*. *thaliana*, the helicase RTEL1 acts in a pathway parallel to the nuclease MUS81 during intrastrand crosslink repair, as demonstrated by hypersensitivity against *cis*-Pt in *rtel1-1* mutants [25,26]. Additionally, RTEL1 was shown to be involved in telomere homeostasis in the absence of the functional telomerase TERT [25]. In contrast to mammalian cells, loss of *Arabidopsis* RTEL1 alone does not lead to telomere loss. The 3’ ends of telomeres are protected by forming D-loop structures (T-loops) to prevent inappropriate recombination between repetitive sequences and chromosome entanglement [27]. In contrast to other eukaryotes T-Loops in *Arabidopsis* might only be present in one but not the other end of the chromosomes [28]. However, T-loops could represent a potential threat to genome stability and must be dismantled to permit efficient telomere replication. Mammalian RTEL1 maintains secondary structures such as T-loops and G-quadruplexes at telomeres [29].

In this study, we aimed to define the role of the RMI2 homologue of *Arabidopsis* in DNA repair and somatic and meiotic HR, as well as its relationship to MUS81 and RTEL1. Surprisingly, we found that both RMI2 and RTEL1 have a previously not characterized function preserving the stability of the 45S rDNA copy number.

## Results

### Characterization of a T-DNA insertion and a CRISPR/Cas induced RMI2 mutant in *A*. *thaliana*

The stability and stimulation of the human Holliday Junction dissolvasome are facilitated by the RMI1 sub-complex containing the HsRMI1 and HsRMI2 proteins [7,8]. In this context, Xu et al. (2008) predicted a plant orthologue for human RMI2 (NP_201159) by BLASTP analysis using the NCBI NR (non-redundant protein sequences) database. Recent proteome analysis demonstrated that the postulated protein is indeed associated with the three other complex partners in *Arabidopsis* [20].

The *Arabidopsis* homologue *RMI2* (At1g08390) contains three exons and two introns, with a length of 884 bp and an open reading frame that codes for a small protein of 137 amino acids.

To characterize the function of RMI2 in *Arabidopsis*, we analyzed the phenotype of the T-DNA insertion mutant At*rmi2-1* (GABI_148E03) [30], with a multi-copy T-DNA insertion in intron II flanked on both sides by LB structures and a 2 bp duplication of chromosomal sequence (S1 Fig). An absence of complete mRNA of *RMI2* in the At*rmi2-1* was demonstrated by quantitative real-time PCR (S2 Fig). To obtain a second independent mutant, we used CRISPR/Cas9-mediated mutagenesis, as recently described [31], to establish the mutant At*rmi2-2*. The target sequence for the Cas9 nuclease was located after nucleotide 67 in exon I of the *RMI2* gene (protoscpacer 5‘ TCTCAGAACGCCGCCTCCCT 3‘). In exon I, At*rmi2-2* harbors a 297 bp insertion originating from the C-terminal portion of the plastid-coded large subunit of RUBISCO (AtCg00490) which was identified by high resolution melting analyses and Sanger sequencing in plants from the second generation after transformation of Col-0 plants (S1 Fig). The respective mutation was validated by cDNA sequencing, which confirmed the insertion resulting in a premature stop codon in the *RMI2* open reading frame (S1 Fig).

### AtRMI2 suppresses homologous recombination in somatic cells

**Fig 1:**
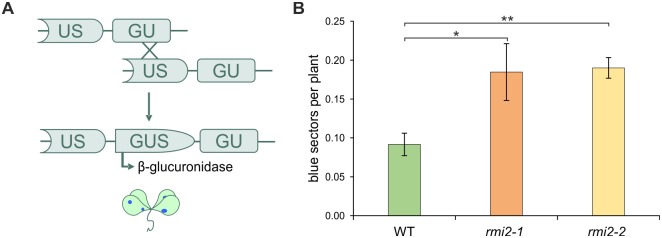
Recombination frequency in somatic cells. (A) Schematic structure of the reporter construct IC9C used for crossbreeding with insertion mutants. An interrupted gene coding for the β-glucuronidase (*GUS*) with overlapping homologies, can be restored by interchromosomal HR. Each restoration is detectable by histochemical staining. (B) Mean recombination frequencies of mutants At*rmi2-1* and At*rmi2-2* are increased by 2-fold compared to wild-type plants (n = 3). Significant differences were calculated using the two-sample *t*-test, two-sided with no equal variance, p-values: *P ≤ 0.05; **P < 0.01.

The suppression of CO products is essential for genomic stability in somatic cells. The RTR complex is one protein complex involved in the maintenance of genomic integrity in somatic tissue [2,6,32,33]. In *Arabidopsis thaliana*, the T-DNA insertion mutants of the corresponding complex partners AtRECQ4A, AtTOP3α and AtRMI1 display elevated recombination frequencies [12,15]. To investigate a putative function of AtRMI2 in the suppression of somatic HR, the mutants At*rmi2-1* and At*rmi2-2* were both crossed with the reporter line IC9C, which harbors a recombination substrate to detect recombination events *in planta* [34]. An interrupted gene coding for the β-glucuronidase (*GUS*) with overlapping homologies, can be restored by interchromosomal HR using the sister chromatid or the homologous chromosome (Fig 1). The recombination frequencies of plantlets harboring a defect in RMI2 was elevated 2-fold compared to wild-type plantlets, which could be demonstrated for the T-DNA insertion mutant *rmi2-1* as well as the Cas9-induced mutant *rmi2-2* (Fig 1). Thus, AtRMI2 appears to be involved in the suppression of HR, leading to a hyper-recombination phenotype if defective.

### AtRMI2, AtRTEL1 and AtMUS81 act in parallel pathways during intrastrand CL repair

**Fig 2:**
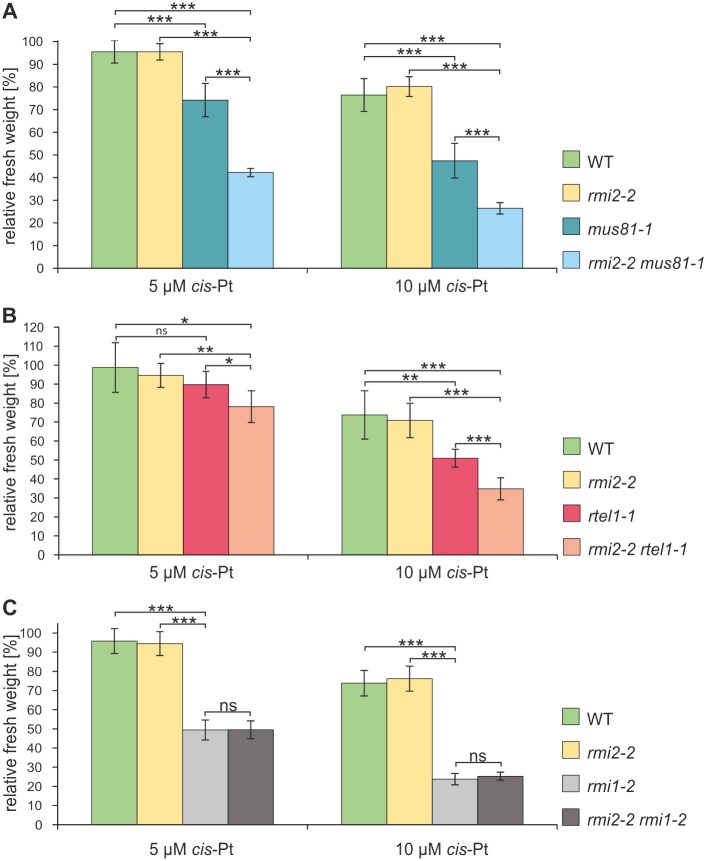
Epistasis analysis of RMI2, MUS81 and RTEL1 in intrastrand CL damage. Relative fresh weights after treatment with 5 or 10 μM *cis*-Pt to induce intrastrand CL DNA damage are depicted (A) The double mutant *rmi2-2 mus81-1* exhibits synergistic hypersensitivity compared to both the single mutants and wild-type plants for all tested concentrations (n = 6). (B) The double mutant *rmi2-2 rtel1-1* exhibits synergistic hypersensitivity compared to both the single mutants and wild-type plants (n = 6). (C) A defect in RMI2 and RMI1 displayed similar hypersensitivity in the double mutant At*rmi2-2 rmi1-2* compared to At*rmi1-2* (n = 8). Significant differences were calculated using the two-sample *t*-test, two-sided with no equal variance, p-values: *P ≤ 0.05; **P < 0.01; ***P < 0.001, ns = not significant.

Persistent DNA lesions induced by DNA-damaging agents can lead to stalled replication forks, which compromise genome stability and cause cell death. To investigate whether RMI2 is involved in the processing of chemically stalled replication forks, we detected hypersensitivity against intrastrand crosslinks (CL) in mutant plants. Plantlets of the mutants *rmi2-1* and *rmi2-2* were cultivated for one week under axenic conditions before being treated with different concentrations of *cis*-Pt, which mainly causes intrastrand CLs [35]. Following two weeks of incubation, the results were obtained by measuring the fresh weight of the plantlets. Analysis was carried out in relation to an untreated control and compared to fresh weights of equally treated wild-type plants. Both mutants, neither *rmi2-1* nor *rmi2-2* exhibited hypersensitivity after treatment with different concentrations of *cis*-Pt (S3 Fig). In previous studies, it was demonstrated that the repair of intrastrand CL is also maintained by the DNA helicase RTEL1 and the nuclease MUS81 operating in pathways parallel to the RTR complex to support genomic integrity [25,26]. We speculated that RMI2 might stabilize the RTR complex, which might allow the remaining complex partners to perform DNA repair reactions with reduced efficiency in the absence of RMI2. Therefore, a role of the protein in DNA repair might only be detectable in the absence of parallel pathways. To test this hypothesis in relationship to the helicase MUS81, we established the double mutant *rmi2-2 mus81-1* by crossbreeding the respective single mutants. Hypersensitivity was analyzed by treatment with *cis*-Pt. The loss of RMI2 along with MUS81 resulted in a synergistic effect (Fig 2). The double mutant *rmi2-2 mus81-1* exhibited a significant increase in sensitivity compared to equally treated wild-type plants and single mutants. Additionally, we analyzed the influence of RMI2 on intrastrand CL repair in relation to the helicase RTEL1 by treating the double mutant *rmi2-2 rtel1-1* with *cis*-Pt. Plants with a defect in RMI2 and RTEL1 exhibited a strong hypersensitivity against *cis*-Pt treatment compared to the respective single mutants and wild-type plants (Fig 2). Thus, we demonstrated that RMI2 has a function in intrastrand CL repair that is independent of RTEL1 and MUS81. Additionally, the double mutant At*rmi2-2 rmi1-2* was established as a control. Here, the double mutant displayed a comparable increase in sensitivity as the At*rmi1-2* single mutant, indicating as expected that RMI2 and RMI1 are part of the same intrastrand crosslink repair pathway (Fig 2).

### AtRMI2 is involved in the repair of aberrant replication intermediates in root meristems independently of AtRTEL1 and AtMUS81

**Fig 3:**
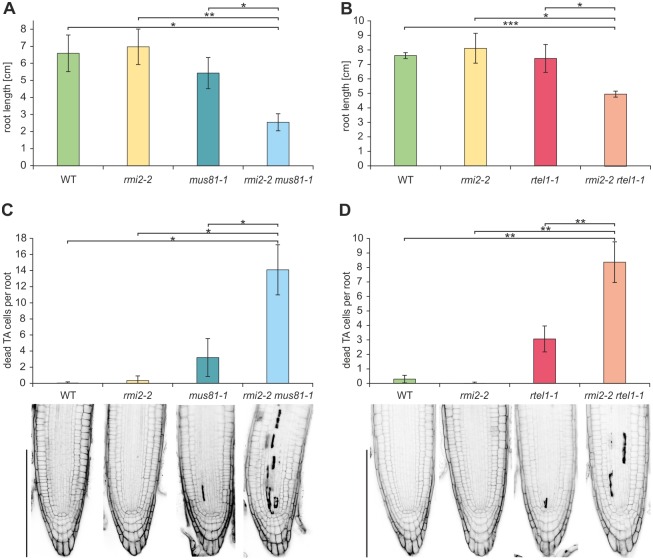
Epistasis analysis of RMI2, MUS81 and RTEL1 as determined by cell death and root length analyses. The relationship of RMI2, MUS81 and RTEL1 in the maintenance of replication-associated repair processes was determined by cell death analysis in root tips and by root length measurement (n = 3). A defect in RMI2 did not lead to any dead TA cells or reduced root length compared to wild-type plants. (A + B) Root lengths in both double mutants *rmi2-2 mus81-1* and *rmi2-2 rtel1-1* were reduced compared to the corresponding single mutants and wild-type plants. (C + D) The number of dead TA cells adjacent to the stem cell niche in the root meristems was significantly elevated in both double mutants *rmi2-2 mus81-1* and *rmi2-2 rtel1-1* compared to the corresponding single mutants and wild-type plants (n = 3). Significant differences were calculated using the two-sample *t*-test, two-sided with no equal variance, p-values: *P ≤ 0.05; **P < 0.01; ***P < 0.001.

As we demonstrated the role of RMI2 in supporting genomic integrity as part of the repair mechanism in intrastrand crosslink damage, we further investigated whether RMI2 might be involved in replication-associated repair processes in general. Therefore we analyzed cell division in dividing tissues of the root meristem. Dividing cells directly influence root growth, and the accumulation of cell damage can result in cell death [36]. Therefore, the root meristem is suitable for analysis of the consequence of defective repair proteins on cell division in general. First, we examined the involvement of RMI2 in ensuring proper root growth, by analyzing the root lengths of the *rmi2-2* mutant line. We measured the root length after 10 days of cultivation by digital root tracking analysis [37]. Plants with a defect in RMI2 were not defective in root growth compared to wild-type plants. However, the additional defect of the nuclease MUS81 leads to severely reduced root length in the respective double mutants compared to both single mutants *rmi2-2* and *mus81-1* and wild-type plants (Fig 3). A similar effect was observed in the double mutant *rmi2-2 rtel1-1*, demonstrating a synergistic effect (Fig 3). Thus, RMI2 appears to be involved in preservation of genomic stability in dividing tissues in a pathway parallel to RTEL1 as well as MUS81, thereby ensuring proper root growth. For a thorough analysis of root meristems, we additionally analyzed the respective mutant lines after 5 days of cultivation with propidium iodide (PI) staining. PI is only able to stain dead cells and is excluded from living cells. Additionally, the root architecture can be visualized by stained intercellular spaces. The root meristem inherits the root stem cell niche (SCN) close to the root tip. Adjacent to the stem cell niche is a region of transiently amplifying (TA) cells, which arise from cell divisions in the SCN [38]. A microscopic examination was carried out by quantifying the dead cells in the TA cell region. Again, the *rmi2-2* single mutant did not reveal an accumulation of dead cells. However, the reduced root growth in the double mutants was accompanied by increased numbers of dead TA cells in *rmi2-2 mus81-1* and *rmi2-2 rtel1-1* compared to each single mutant *mus81-1*, *rtel1-1* and wild-type plants (Fig 3). The accumulation of DNA lesions might lead to replicative catastrophe and cell death. As we were able to show previously with this assay that RTEL1 and MUS81 act in parallel pathways as shown by the synergistic phenotype of the double mutant [25], we can now conclude that RMI2 acts parallel to both RTEL1 and MUS81 and is important for genomic stability in dividing tissues.

### AtRMI2 is not involved in late meiotic processes

The RTR complex partners AtRMI1 and AtTOP3α have unique functions in meiotic recombination, as respective mutant plants suffer severe chromosomal breakage, resulting in meiotic arrest and sterility [14,15]. In contrast, no similar defect was reported for AtRECQ4A [12,15,39]. Thus, a specific sub-complex consisting of AtRMI1 and AtTOP3α with unique functions seems to be present in plants. It was important to test whether RMI2 was required for the functionality of this sub-complex in *Arabidopsis*, since data from mammalian studies showed that a sub-complex of RMI1 and RMI2 is formed before interaction with TOP3α [7,8]. Both RMI2 mutants were fully fertile, and analysis of meiotic progression by DAPI-stained chromatin spreads did not show any defects during the segregation of chromosomes (S4 Fig). Thus, RMI2 is not required for the specific functions of RMI1 and TOP3α in a later step of meiotic recombination.

### Loss of AtRMI2 and AtRTEL1 leads to severe fertility defects

**Fig 4:**
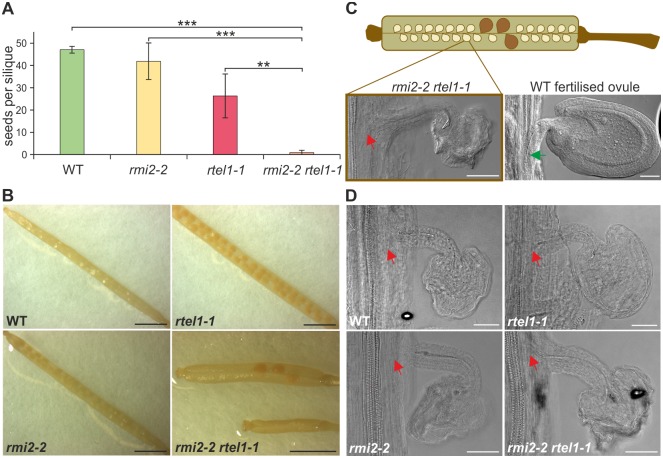
Fertility defect due to simultaneous loss of RMI2 and RTEL1. (A) The amount of seeds per silique was dramatically reduced in *rmi2-2 rtel1-1* double mutants compared to single mutants and wild-type plants (n = 5). (B) Photographs of siliques from each genotype are depicted. Bar = 2 mm. (C) Schematic representation of a silique from the double mutant *rmi2-2 rtel1-1* exhibiting a large amount of undeveloped ovules. An exemplary ovule of *rmi2-2 rtel1-1* is depicted and the red arrow illustrates the missing connection to the vascular system. Additionally a fertilised wild-type ovule is depicted with a connected vascular system as indicated by a green arrow. Bar = 50 μm. (D) Ovules from demasculated plants of each genotype with artificially inhibited fertilization resemble ovules from mature *rmi2-2 rtel1-1* siliques. The red arrow illustrates the missing connection to the vascular system. Bar = 50 μm. Significant differences were calculated using the two-sample *t*-test, two-sided with no equal variance, p-values: **P < 0.01; ***P < 0.001.

We made a surprising discovery while analyzing the *rmi2-2 rtel1-1* double mutant. We observed a major deficiency in the fertility of plants with a defect in RMI2 along with RTEL1. In this context, we did not observe a similar defect in the double mutant *rmi2-2 mus81-1*. Fertility was determined by counting seeds per silique and comparing siliques from the double mutant *rmi2-2 rtel1-1* with siliques from the single mutants and wild-type plants. The amount of seeds was severely decreased in *rmi2-2 rtel1-1* leading to a synergistic effect compared to *rmi2-2*, *rtel1-1* and wild-type siliques. This was rather unexpected as the *rmi2-2* mutant was not defective in progression through meiosis and decreased fertility of *rtel1-1* had not been described thus far. We first analyzed the rarely produced seeds in *rmi2-2 rtel1-1* by embryo preparation and microscopic examination. The embryos of the respective seeds were not affected and developed like wild-type embryos (S5 Fig). However, next to single seeds in the siliques, we observed a high number of undeveloped ovules. The vascular strand of the respective ovules was not connected to the vascular network of the carpel (Fig 4). This is characteristic of unfertilized ovules in *Arabidopsis* [40]. To validate the assumption of a fertilization defect in *rmi2-2 rtel1-1* double mutants, we conducted a demasculation experiment by excising the anthers from four-week-old plants of the double mutant as well as single mutants and wild-type plants. We compared the resulting unfertilized ovules from each plant line to the observed phenotype from the mature siliques of *rmi2-2 rtel1-1*. The ovules of artificially unfertilized flowers resembled the ovules in *rmi2-2 rtel1-1* and indicate a potential defect in fertilization.

### AtRMI2 and AtRTEL1 are essential for the development of the male gametophyte

**Fig 5:**
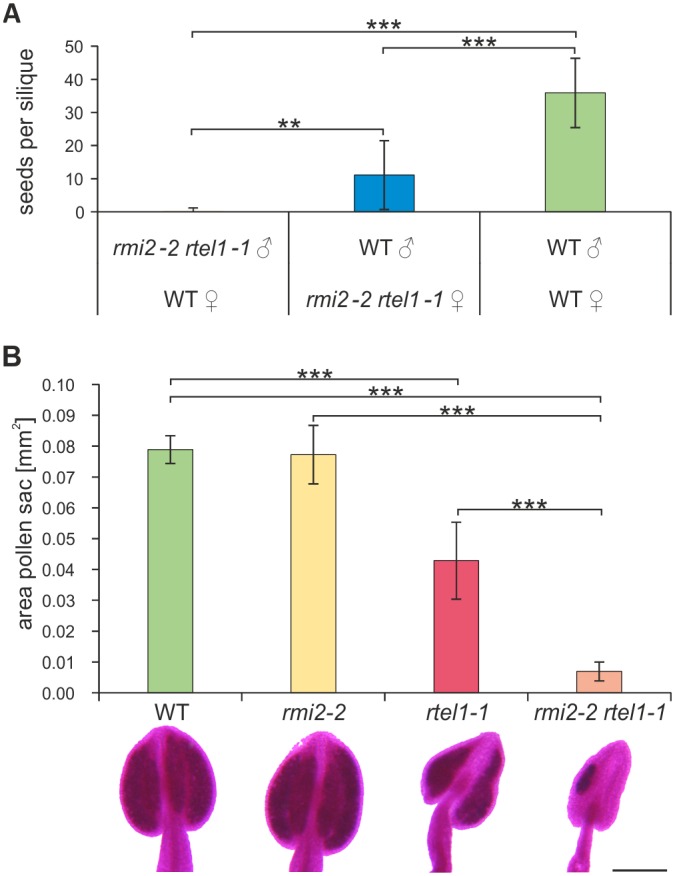
Impaired male germline development due to simultaneous loss of RMI2 and RTEL1. (A) By crossing *rmi2-2 rtel1-1* with wild-type plants (n = 12), practically no seeds could be obtained using pollen from *rmi2-2 rtel1-1* mutants for crossbreeding. Alternatively, crossbreeding with wild-type pollen led to a significant restauration of seed numbers. The fertility defect was mainly confined to the male germline. (B) The amount of pollen found in *rmi2-2 rtel1-1* anthers, derived from anther area measurement was drastically decreased compared to single mutants and wild-type plants (n = 20). Nevertheless, Alexander staining revealed viable pollen in all genotypes without exceptions. Exemplary stained anthers of all genotypes are depicted. Bar = 200 μm. Significant differences were calculated using the two-sample *t*-test, two-sided with no equal variance, p-values: **P < 0.01; ***P < 0.001.

Fertilization is necessary for embryo and endosperm development within the seeds of flowering plants and is carried out by male and female gametes. The plant germline can be considered from the archesporial cells, which give rise to micro- and megaspore mother cells contributing to the gametophyte development [41]. To determine whether the defect might be specific to the male or female germline, a backcross experiment was carried out using the mutant line *rmi2-2 rtel1-1* as either the female or male partner in a crossbreeding with wild-type plants. Wild-type plants fertilized with pollen from plants with a defect in RMI2 and RTEL1 hardly produced offspring (Fig 5). In contrast, *rmi2-2 rtel1-1* mutant lines fertilized by wild-type pollen developed a substantial amount of progeny compared to a wild-type control crossing. Therefore, the fertility defect in *rmi2-2 rtel1-1* is more severely pronounced in the male germline development.

To examine this observation further, we analyzed male meiosis by examination of chromosome spreads from pollen mother cells (microspore mother cells, PMCs) undergoing meiosis using fluorescence microscopy [42]. Intriguingly, the number of PMCs extracted from flower buds of one inflorescence was dramatically decreased in *rmi2-2 rtel1-1* to that observed in chromosome spreads from wild-type flowers. On average more than 30 times less PMCs could be observed in the double mutant *rmi2-2 rtel1-1* compared to the WT (S2 Table, p-value = 0,034, n = 4).

Furthermore, we examined the viability of pollen within anthers by Alexander staining [43]. We did not observe nonviable pollen in either the double or the single mutants (Fig 5). However, by digital measurement of the area of the pollen sac oriented with the connective in the middle of each anther, we observed an extremely reduced amount of pollen in *rmi2-2 rtel1-1* compared to *rmi2-2* and *rtel1-1*. The reduced amount of pollen in *rmi2-2 rtel1-1* reflected the extremely reduced number of PMCs. So far we assumed a potential defect affecting genome stability in the dividing tissue of the floral organ before entry into meiosis.

### AtRMI2 acts in a pathway parallel to RTEL1 in the suppression of homologous recombination

**Fig 6:**
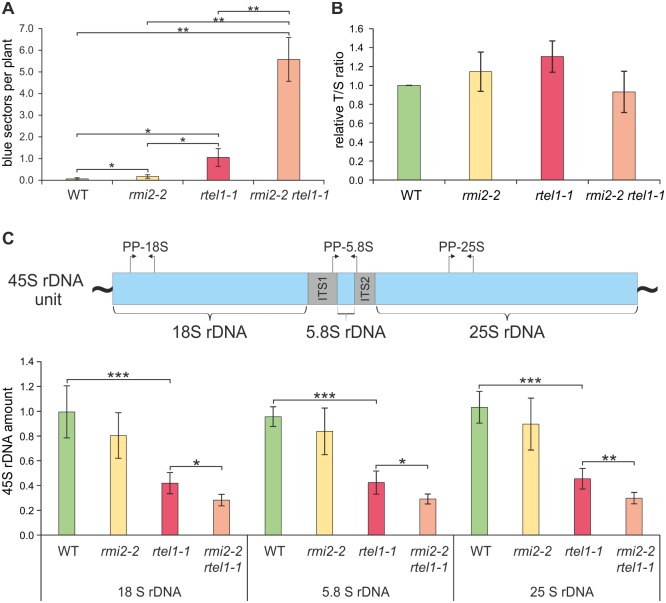
Loss of 45S rDNA repeats due to simultaneous defects in RMI2 and RTEL1. (A) Genomic instability is associated with elevated recombination frequency, which was observed for *rmi2-2 rtel1-1* mutants (n = 5). A tremendous 80-fold increase was shown for the double mutant *rmi2-2 rtel1-1* compared to wild-type plants. (B) The length of telomeres was determined by the ratio of telomeric repeats to a single copy gene (T/S ratio). No statistically significant difference could be detected between wild-type and mutant plants (n = 3). (C) The double mutant *rmi2-2 rtel1-1* exhibits a significant loss of 45S rDNA repeats compared to the single mutants and wild-type plants. Copy numbers of 18S, 5.8S and 25S rDNA sequences were analyzed independently with primer pairs as indicated in the schematic overview of one 45S rDNA unit (n = 6). Significant differences were calculated using the two-sample *t*-test, two-sided with no equal variance, p-values: *P ≤ 0.05; **P < 0.01; ***P < 0.001. PP = primer pair, ITS = internal transcribed spacer.

Tight regulation of HR is important for genomic stability. The RTR complex as well as the helicase RTEL1 play crucial roles in the suppression of HR in somatic cells. We demonstrated that AtRMI2 participates in the suppression of hyper-recombination. As we postulated that genomic instability in somatic cells is a source of defects in male germline development, we tested the spontaneous recombination frequency in plants with a defect in RMI2 along with RTEL1 (Fig 6). To measure recombination frequency, we analyzed two-week-old seedlings of the single mutants and *rmi2-2 rtel1-1* after histochemical staining with the β-glucuronidase substrate. The absolute number of blue sectors per plant was dramatically increased in *rmi2-2 rtel1-1* (Fig 6). We observed an extreme hyper-recombination phenotype in plants defective for RMI2 and RTEL1 that was elevated by almost two orders of magnitude (80-fold) compared to wild-type plants. Thus, repetitive genomic sequences would be severely destabilized in the double mutant, most likely causing serious genomic instability.

### Quantitative real-time PCR analysis demonstrates that 45S rDNA repeats but not telomeres are targets for the suppressors AtRMI2 and AtRTEL1

Prominent repetitive sequences in eukaryotic genomes are found at telomeres and within the 45S rDNA gene clusters. Therefore, we decided to test the stability of both classes of sequences in the double mutant. In a previous study, we demonstrated the involvement of RTEL1 in telomere homeostasis in *A*. *thaliana* [25]. A defect in RTEL1 worsened the gradual telomere shortening in plants defective for the telomerase TERT. Development of the double mutant *rtel1-1 tert* is arrested after the fourth generation instead of the tenth generation, as in *tert* single mutants [25,44]. RTEL1 is proposed to be involved in the destabilization of D-loop structures (T-loop) at telomeres to support replication at chromosome ends. It was tempting to speculate that telomere instability might be the cause of the accumulation of cell damage leading to sterility in *rmi2-2 rtel1-1*. Therefore, we examined the length of telomeres in *rmi2-2 rtel1-1* compared to *rtel1-1* and *rmi2-2* by MMQPCR (monochrome multiplex quantitative PCR) [45]. MMQPCR was performed with degenerate *A*. *thaliana*-specific telomeric primers as well as primers to amplify a single copy sequence as the DNA quantity reference. The relative ratio of telomeric and single copy products (T/S ratio) was determined after calculation of DNA concentration using the standard curve, generated from a serial dilution of DNA of known concentration and normalization to the wild-type T/S ratio. Telomeres in plants with a defect in RTEL1 were not affected as shown by similar T/S ratios in *rtel1-1* and wild-type plants after 4 weeks of cultivation, and this was in agreement with previous reports [25,26]. The single mutant *rmi2-2* as well as the double mutant *rmi2-2 rtel1-1* were also not impaired in telomere length compared to wild-type plants (Fig 6). Therefore, telomeres were not destabilized in the double mutant.

Next, we examined the stability of ribosomal DNA (rDNA) repeats. The 45S rDNA codes for the ribosomal 18S, 5.8S and 25S rRNAs and is located on chromosome II and IV in *A*. *thaliana*. Organized in tandem repeats consisting of units coding for one copy of 18S, 5.8S and 25S rDNA each, the 45S rDNA comprises 3.5 to 4 megabases [46]. We measured the amount of 45S rDNA repeats using quantitative real-time PCR independently targeting 18S rDNA, 5.8S rDNA and 25S rDNA. We observed a significantly reduced amount of all three rDNA coding sequences in plants defective for RTEL1. This was surprising, as such a phenotype has not been reported to our knowledge for other eukaryotes before. The amount of rDNA repeats in *rmi2-2* mutants was stable, but the double mutant *rmi2-2 rtel1-1* suffered a synergistic loss of all three rDNA coding sequences compared to the single mutants and wild-type plants (Fig 6). In the double mutant, we estimated that approximately 70% of 45S rDNA repeats are lost compared to wild-type. Thus, the dramatically enhanced HR efficiency is indeed correlated with massive instability of the 45S rDNA repeats in the double mutant.

### Cytological analysis confirms that AtRMI2 and AtRTEL1 are both acting independently to suppress 45S rDNA repeat instability

**Fig 7:**
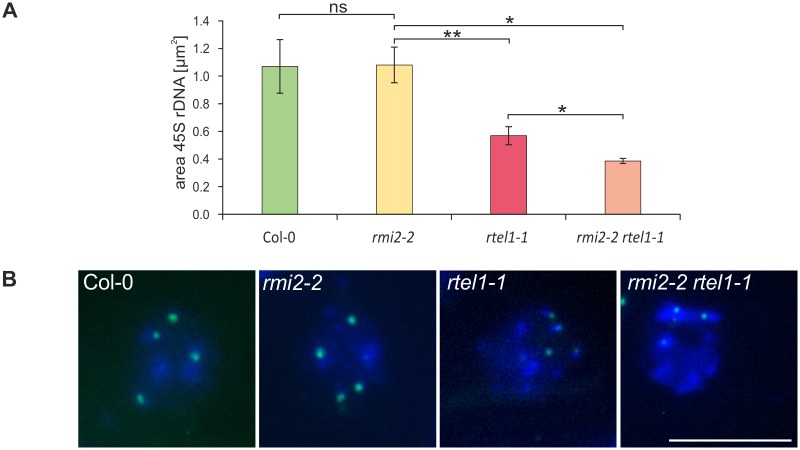
Quantification of the decreased amount of 45S rDNA repeats in *rmi2-2 rtel1-1* by FISH analyses. (A) Quantification of the amount of 45S rDNA was significantly reduced in *rmi2-2 rtel1-1* compared to the respective single mutants and wild-type plants, as determined by FISH analyses with an Atto488 labelled 45S rDNA probe and DAPI staining (n = 3). (B) Additionally, an exemplary nucleus of each genotype is depicted, with four rDNA areas (green) on the chromatin (blue) of interphase nuclei (Scale bar = 10 μm). Significant differences were calculated using the two-sample *t*-test, two-sided with no equal variance, p-values: *P ≤ 0.05; **P < 0.01; ns = not significant.

To sustain the unexpected and surprising decrease in copy numbers of the 18S, 5.8S and 25S rDNA sequences by quantitative real-time PCR, we further examined the area of 45S rDNA directly on chromatin from nuclei of the double mutant *rmi2-2 rtel1-1*, the respective single mutants and wild-type plants. Therefore a cytological approach with hybridization of fluorescent probes (fluorescence *in situ* hybridization, FISH) was applied. Quantification of fluorescence signals with a 45S rDNA probe and chromatin staining by DAPI was carried out with whole inflorescences from 4 week old plants. The amount of 45S rDNA repeats were determined by measurement of the area of the fluorescence signals with an Atto488 labeled probe against 45S rDNA in three independent chromatin spreads. For every chromatin spread containing three inflorescences, a minimum of 20 somatic nuclei were analyzed. We could validate a significant reduction in 45S rDNA signals in the double mutant *rmi2-2 rtel1-1* compared to *rtel1-1*, *rmi2-2* and wild-type plants with this approach (Fig 7). Plants with a defect in RMI2 display the same amount of rDNA signals as wild-type plants, but *rtel1-1* mutants already show a pronounced decrease to about 60% in 45S rDNA repeat signals and the double mutant is even stronger affected with a decrease to about 42%. Both RMI2 and RTEL1 independently support the stability of 45S rDNA repeats in somatic tissue from whole inflorescences (Fig 7).

## Discussion

The structural protein RMI2 is an integral component of the human BTR complex and important for genomic stability [7,8]. Here, we report that the RMI2 homologue of *Arabidopsis*, in addition to its conserved roles in DNA repair, has an essential function in concert with the helicase RTEL1 in the suppression of 45S rDNA repeat loss to preserve genome stability in plants. Both factors facilitating two independent pathways to support the maintenance of copy numbers of these repetitive elements.

### The role of RMI2 in DNA repair and HR

We demonstrated an increased recombination frequency in plants depleted of RMI2. Both mutant plant lines *rmi2-1* and *rmi2-2* exhibit significantly elevated frequencies of spontaneous HR events compared to wild-type plants. Thus, AtRMI2 is involved in the suppression of somatic HR. The formation of sister chromatid exchange by HR is postulated to be suppressed by processing of recombination intermediates via the dissolution pathway that results in repair products without CO formation [2]. Different mammalian cell lines exhibit elevated rates of SCE if RMI2 is depleted. Similar to our findings for AtRMI2, it has been shown before that loss of any of the other three members of the RTR complex, namely AtRECQ4A [12], AtTOP3α, and AtRMI1 [15], also leads to hyper-recombination. This is a strong indication for functional conservation between plants and animals and applies to all four partners of the respective RTR and BTR complexes. Next to the function of RMI2 in HR, we demonstrated a role for RMI2 in intrastrand crosslink repair. We were able to elucidate a function for AtRMI2 in repair of *cis*-Pt induced intrastrand crosslinks only in the absence of the nuclease MUS81 or the helicase RTEL1. Additionally, we could confirm that RMI1 and RMI2 are involved in the same pathway during intrastrand crosslink repair. This indicates that, as in mammals, AtRMI2 plays a stabilizing role for the RTR complex, which is necessary to repair these lesions independently of MUS81 and RTEL1. Therefore, in principle, loss of AtRMI2 is expected to cause defects in DNA repair that are less severe compared to the loss of the other partners of the RTR complex. Indeed, much stronger phenotypes have been reported for double mutants where AtRECQ4A instead of AtRMI2 was mutated. A synthetically lethal phenotype was observed in *recq4A-4 mus81-1* double mutants [24]. Thus, the defect in processing of replication intermediates is much more severe in the absence of RECQ4A than in the absence of RMI2. Similarly, plants with a defect in RTEL1 together with RECQ4A suffer severe growth defects [25,26]. As we used two different *RMI2* mutants (one T-DNA insertion line and one Cas9-induced knock out line) that both exhibit identical phenotypes in our analysis, we can exclude the possibility that the weaker phenotype of RMI2 with respect to *recq4A-4* is due to partial enzyme activity of the RMI2 protein in the respective mutants. In the current study, we used the weaker phenotype to our advantage: only by working with At*RMI2* mutant plants, we were able to define which cells are most sensitive to the DNA damage that results when the RTR complex is not performing properly. Moreover, due to lethality or massive growth defects in the respective double mutants, no epistasis analysis can be performed with other complex partners such as RECQ4A. Apart from intrastrand crosslink repair and suppression of HR, RMI2 is involved in the maintenance of replication intermediates in root meristems. The root meristem is a highly dividing tissue, in which cellular processes such as replication need to be maintained thoroughly to preserve genomic stability. Genomic instability, due to blocked replication fork progression and persistent DNA lesions, can lead to cell cycle delay and might result in programmed cell death. We observed a significant increase in dead cells in *A*. *thaliana* root meristems when RMI2 along with other repair factors, such as MUS81 or RTEL1 were depleted. The double mutant *rmi2-2 mus81-1* exhibits reduced root length. This phenotype was correlated with a greatly increased number of dead cells in the TA cell region adjacent to the stem cell niche in *rmi2-2 mus81-1*. Further analysis demonstrated a similar phenotype in the double mutant *rmi2-2 rtel1-1*, in which a highly increased number of dead TA cells and reduced root length were observed. Interestingly, defects in RTEL1 and RECQ4A induce a complete loss of root structures, as was recently demonstrated [25,26]. It was proposed that a cell cycle delay could cause the loss of synchronicity during cell division resulting in aberrant root architecture. Additionally, the root growth defect in *rtel1-1* was postulated to be primarily due to the inhibition of cell division rather than cell elongation [26]. Here, we demonstrated that RMI2, as an RTR complex partner, is involved in the removal of aberrant replication intermediates independently of the nuclease MUS81 and the helicase RTEL1 in meristematic tissues.

### RMI2 together with RTEL1 are required for male fertility in *Arabidopsis*

We could not find any indication for a function of RMI2 in late meiotic recombination, which was shown for its sub-complex partner RMI1 and the topoisomerase TOP3α [14,15]. The meiosis-specific functions of RMI1 and TOP3α are also independent of the RecQ helicase RECQ4A [12,15,39]. The most plausible hypothesis would be that an RTR sub-complex containing AtTOP3α and AtRMI1 exists and that it is involved in the dissolution of meiotic recombination intermediates. Although no indication for a comparable role of the BTR complex in mammals in processing of late meiotic recombination has been reported so far, it is tempting to speculate that the functional conservation of the RTR complex also applies for meiotic recombination.

Intriguingly, we observed a severely increased fertility defect in plants defective for RMI2 along with RTEL1. Fertility analysis in *rmi2-2 rtel1-1* demonstrated significantly decreased amounts of seeds per silique. Additionally, we observed a fertility defect in *rtel1-1*. The analysis of chromatin spreads extracted from inflorescences of the insertion mutants *rtel1-1* and *rmi2-2* did not reveal any damage in progression through meiosis. However, the double mutant *rmi2-2 rtel1-1* inherits a tremendously decreased number of PMCs, compared to the number of PMCs isolated from wild-type inflorescences. An analysis of embryo development of the very rarely produced seeds did not reveal any irregularities compared to wild-type seeds. However, we observed a high amount of unfertilized ovules, and further analysis of pollen viability revealed a highly decreased amount of pollen in *rmi2-2 rtel1-1* mutant plants. Taken together, it is highly likely that the fertility defect in *rmi2-2 rtel1-1* is due to defects in the premeiotic cell line development. The defect was much more severe for the male germline than for the female germline, which differ by the number of cell divisions necessary for gametophyte development [47,48]. We hypothesized that the fertility defect might be caused by increased genomic instability during premeiotic cell divisions within the sporophytic male floral organs during sporogenesis as we could define the defect occurring before entry into meiosis. The sporogenesis in flowering plants is characterized by differentiation of hypodermal cells within the floral organs into microsporocytes and megasporocytes [47]. Accumulating DNA lesions could lead to replicative stress, which could result in cell death. For the production of the required number of PMCs, male archesporial cells undergo multiple divisions, and the male germline would be especially vulnerable to such a defect. This is in contrast to the female archesporial cell in each ovule, which does not undergo mitotic divisions but instead directly functions as a megaspore mother cell [47,48].

### RMI2 and RTEL1 suppress the instability of 45S rDNA by different pathways

We hypothesized that the source of this fertility defect originated from the accumulation of replicative damage particularly during cell divisions throughout anther development. Insufficient somatic repair processes could result in either the slowing down or blockage of premeiotic cell divisions and eventually programmed cell death. Alternatively, cell cycle regulation might also hinder mitotic nuclei to progress into meiosis. In nematodes, it has been demonstrated that a mutant allele of *spar-1* (RTEL1 homologue) exhibits a 3-fold increase in germline apoptosis [49]. Moreover, in *C*. *elegans*, the Bloom syndrome homologue *him-6* (RECQ4A homologue) and *top-3* are required to maintain genome stability in the germline [50].

The regulation of D-loop formation plays an important role in the regulation of HR. RTEL1 is known to be important for disrupting D-loops and reversing HR processes [49]. A similar function was proposed for the RTR complex via its helicase function [1]. Alternatively, the HR-suppressing role of the RTR complex was attributed to its dissolution function, which processes recombination intermediates exclusively to non-crossover products [51]. We speculated that a simultaneous defect of both entities might lead to the accumulation of toxic amounts of D-loop structures or CO products resulting in a replicative catastrophe due to massive hyper-recombination.

Indeed, we demonstrated a highly increased recombination frequency in the *rmi2-2 rtel1-1* double mutant of almost two orders of magnitude (80-fold). This tremendous increase in recombination frequency led us to the assumption that repetitive genomic sequences should be severely destabilized in the double mutant. As RTEL1 is known to be involved in telomere maintenance, we analyzed telomere stability of the respective mutants. Mouse embryonic stem cells depleted of RTEL1 exhibit shorter telomeres [52]. However, mutant plants defective in RTEL1 display identical telomere length compared to wild-type plants [25,26]. We did not observe a defect in telomere homeostasis in either the single mutants *rmi2-2* and *rtel1-1* or the double mutant *rmi2-2 rtel1-1*. It has been shown before for *Nicotiana tabacum*, that recombination is downregulated at telomeres by the folding into a compact nucleoprotein structure [53]. Additionally, suppression of DNA damage signaling was reported for telomeres, suggesting that hyper-recombination is not as severe on this kind of repetitive element [54]. However, another prominent repetitive element in eukaryotic genomes are the 45S rDNA tandem repeats. *Arabidopsis* 45S rDNA genes are localized on chromosomes II and IV. *Arabidopsis* 45S rDNA genes are found to be organized in the NOR regions and code for the catalytic cores of the ribosomes, which are the protein-synthesizing machines of the cell [46,55,56]. Appropriate protein machinery is essential for the viability of cells, emphasizing the thorough maintenance of rDNA genes. It was published recently that only one of both NOR regions is active in *Arabidopsis*, reducing by one-half the number of rDNA genes available to accomplish the cellular demands for protein synthesis [57].

It was previously shown that plants with a defect in the chromatin assembly factor (CAF1) exhibit progressive loss of 45S rDNA and telomeres and suffer abnormal morphology and disorganized meristems. Additionally, the respective mutants display an elevated rate of recombination and strongly affected fertility as well as viability [58–61]. The loss of 45S rDNA is dependent on homology-dependent repair mechanisms, as an additional defect of the recombinase AtRAD51B decreases the rate of rDNA loss [62]. We showed, for the first time, that a severely decreased amount of rDNA repeats is correlated with the loss of RTEL1 about half of the copies at least for plants. Notably, an even higher loss of tandem repeats in the double mutant *rmi2-2 rtel1-1* to about a third of the copies was observed. Thus, RMI2 is involved in the maintenance of 45S rDNA genes independently of RTEL1. We are convinced that there is a direct correlation between the hyper-recombination phenotype and the loss of 45S rDNA tandem repeats. We assume that due to the fact that in comparison to other organs more cell divisions occur during male germline development, especially severe reduction of rDNA repeats might occur in this tissue. This would result in cells that could not appropriately dissolve the massive amount of aberrant replication intermediates and would, therefore, slow down cell divisions. Such defects might promote cell death and the massive loss of cells in the male germline. Very little is known about rDNA instabilities and phenotypic effects in other eukaryotes. In an elder study the molecularly uncharacterized *D*. *melanogaster* mutant *bobbed* was described, relating a low number of rDNA repeats with severe growth phenotype as well as sterility [63]. It seems to be a general phenomenon that different kinds of genome instabilities might result in various developmental defects in multicellular eukaryotes. For example, it was recently published that impaired polynucleotide kinase–phosphatase (PNKP) causes severe defects in the tissues of the developing nervous system in humans. PNKP modifies DNA breaks during DNA repair, and phenotypes of defective cells indicate the dependency of neurogenesis on proper genome maintenance [64]. Here, we showed that factors suppressing hyper-recombination are essential for the development of the male germline in plants.

## Materials and Methods

### Plant material and growth

For characterization of RMI2, a T-DNA insertion mutant At*rmi2-1* was used (GABI_148E03; [30]). The second insertion mutant At*rmi2-2* was established by Cas9-mediated mutagenesis of *Arabidopsis* wild-type plants (Columbia-0). Cas9-mediated mutagenesis was carried out as described in 31. The generation of double mutants was mediated by crossbreeding with *mus81-1* (GABI_113F11) and *rtel1-1* (Salk_113285; [65]), which have been described previously [24,25]. For the analysis of recombination frequency, mutant lines were crossed to the IC9C reporter line [34]. All plants were either grown in soil in a greenhouse at 22°C with a 16 h light / 8 h dark cycle or under axenic conditions on agar plates containing germination medium (GM, 4.9 g/l Murashige and Skoog, 10 g/l sucrose and 7.6 g/l agar; pH 5.7) in a plant culture chamber with stable conditions of 16 h light at 22°C and 8 h dark at 20°C. For axenic conditions, the seeds were surface sterilized using 4% sodium hypochlorite and stratification at 4°C overnight.

### Sensitivity assay

The sensitivity analysis was performed as previously described [12]. After 7 days of growth under axenic conditions, 10 plantlets were transferred to each well of a six-well plate containing either 5 ml of liquid GM for the untreated control or 4 ml of liquid GM for treatment with a genotoxic agent. The respective concentration of the genotoxic agent was dissolved in 1 ml of liquid GM and added after an additional day of growth. The fresh weights of the plants from each well were measured after 13 days of incubation. The fresh weights of the plants after genotoxic treatment were normalized to an untreated control of the same plant line.

### HR assay

Recombination frequency were determined by HR assay as described previously [12]. After 15 days of growth under axenic conditions, 40 plantlets were stained (5 ml/l X-GlcA in 2.5% dimethylformamide, 2 ml/l 1% sodium azide in distilled water, diluted in 100 mM phosphate buffer) for 2 days at 37°C. Extraction of plant pigments after staining procedure was done by exchanging the staining solution with 70% ethanol and incubation at 60°C overnight. Blue sectors on each plantlet were quantified with a binocular microscope.

### Root growth assay

The analysis of root meristems with propidium iodide (PI) staining was performed as previously described [25]. After 4 days of growth under axenic conditions in vertical orientation, roots were transferred to microscopic slides in PI solution (5 μg/ml) and covered with a cover slip for analysis with a confocal laser scanning microscope. For root length measurement, plant growth was extended to 10 days in total. The determination of root length was performed by digital root tracking analysis with the image processing program ImageJ smart root tool [37]. Each experiment was carried out with at least 10 roots.

### Fertility analysis and seed preparation

Plants were grown in soil for approximately 4 weeks to quantify a defect in fertility. To examine the amount of seeds per silique, 5 mature siliques from 5 individual plants were harvested and incubated in 70% ethanol overnight. The number of seeds per silique was determined in closed siliques with a binocular microscope. For microscopic examination of ovules, siliques were dissected along the mid region to remove the carpels. After the dissection of siliques, cleared ovules were incubated in clearing solution (2 g chloral hydrate, 0.25 ml glycerol, and 0.5 ml distilled water) for 1 h. The analysis was performed using optical microscopy with differential interference contrast. Pollen viability was tested by staining anthers from 4-week-old plants by Alexander staining [43]. Whole flower buds were incubated for 1 h in fixative solution (ethanol: chloroform: glacial acetic acid, proportionally 6:3:1). After washing the flower buds with distilled water, anthers were cleared and stages 12–13 [66] were transferred to microscope slides with staining solution (1 ml ethanol (95%), 100 μl Malachite green (1%), 2.5 ml glycerol, 1 ml phenol, 0.5 g chloral hydrate, 500 μl acid fuchsin (1%), 50 μl Orange G (1%), and 400 μl glacial acetic acid in 10 ml distilled water) and incubated at 60°C overnight. Analysis was carried out using a binocular microscope and digital imaging.

### Quantitative real-time PCR

Two different real-time PCR approaches were performed with genomic DNA extracted from separate biological replicates after 4 weeks of cultivation under axenic conditions. The genomic DNA template was combined with 5 μl 2x qPCR Master Mix KAPA SYBR FAST-containing SYBR Green dye (Kapa Biosystems), 0.5 μl of each primer (10 mM) and distilled water in a total volume of 10 μl. The Light Cycler 480 instrument (Roche Diagnostics) was used with a 384-well block system. For MMQPCR (monochrome multiplex quantitative PCR), a concentration of 20 ng of genomic DNA was used together with two primer pairs in a multiplexing system to detect telomere length as previously described in *A*. *thaliana* [45]. Thereby, one *Arabidopsis*-specific primer pair was used for the amplification of telomeres. A second primer pair was used for the amplification of the single copy sequence of the *CYP5* gene for normalization. The analysis was performed with three separate biological replicates. To measure the amount of 45S rDNA copies, a concentration of 0.5 ng of genomic DNA was used together with one primer pair as previously described for *A*. *thaliana* [62]. The amplification of the reference gene Ubiquitin 10 and either 18S rDNA, 5.8S rDNA or 25S rDNA was performed with three technical replicates in separate wells. Sequences of used primer pairs see S1 Table. The analysis was conducted with six separate biological replicates.

### Fluorescence in situ hybridisation (FISH)

For Quantification of 45S rDNA repeats a cytological approach was carried out using whole inflorescences of respective plants and a DNA probe against the rDNA locus with Fluorescence hybridisation, according to protocols described previously [67,68] Somatic chromosomes were prepared from flower material of mutant and wild-type plants, which were grown under the same conditions simultaneously. The analyses of each plant line was performed in parallel using the same aliquot of Atto488 labelled hybridization probe, originating from plasmid pTa71, labelled following manufacturer’s recommendations (Nick Translation Labeling Kit, Jena Bioscience) [69]. All images were acquired using the same parameters and the area of the fluorescence signals were quantified using the imaging software ImageJ with background subtraction and a threshold of 5 standard deviations equally.

### PCR-based genotyping of mutant lines

To determine the genotypes of the T-DNA insertion lines and the Cas9-mediated mutant line, one or two specific primer pairs were used. For the T-DNA insertion lines, the results from one gene-specific primer pair targeting 5’ and 3’ of the T-DNA insertion was combined with a primer pair targeting the gene-specific sequence and a sequence located in the T-DNA region. Genotyping of the T-DNA insertion mutants *rtel1-1* and *mus81-1* was performed as previously described [24,25]. For genotyping of the T-DNA insertion line *rmi2-1* see S1 Table and for Cas9-mediated insertion line *rmi2-2* one gene specific primer pair was used targeting 5’ and 3’ of the insertion resulting in one or two PCR products (S1 Table).

### Statistical methods

Significant differences were calculated using the two-sample *t*-test, two-sided with no equal variance, p-values: *P ≤ 0.05; **P < 0.01; ***P < 0.001, ns = not significant.

### Accession numbers

The TAIR accession numbers for the sequences of genes used in this study are as follows: *A*. *thaliana* RMI2 (At1g08390), *A*. *thaliana* RTEL1 (At1g20750), and *A*. *thaliana* MUS81 (At4g30870).

## Supporting Information

S1 Fig Gene structure and mutant lines used in this study. (A) Gene structure of the At*RMI2* gene with three exons and two introns and a length of 884 bp. (B) The At*rmi2-1* mutant carries a multi-copy T-DNA insertion in intron II flanked on both sides by LB structures and bordered by a 2 bp duplication of chromosomal sequence. (C) The Cas9-mediated mutant At*rmi2-2* carries a 297 bp insertion in exon I, harbouring a stop codon (red box) in frame, which was validated by cDNA analysis.
(PDF)

S2 Fig At*RMI2* expression analysis in the T-DNA mutant At*rmi2-1*. Absence of the full-length mRNA of *RMI2* in the T-DNA insertion mutant *rmi2-1* was tested by quantitative real-time PCR with three different primer pairs. The expression of the gene 5' from the insertion locus was comparable to wild-type expression. We could not detect a transcript spanning the T-DNA insertion. The expression 3' of the insertion locus was severely decreased.
(PDF)

S3 Fig Sensitivity analysis after crosslink damage in At*rmi2-1* and At*rmi2-2*. Relative fresh weight after treatment with 5 or 10 μM *cis*-Pt to induce intrastrand crosslink DNA-damage are depicted. Both mutant lines, the T-DNA insertion line *rmi2-1* and the Cas9-induced insertion line *rmi2-2* did not show elevated sensitivity compared to wild-type plants.
(PDF)

S4 Fig DAPI stained chromatin of At*rmi2-2* and wild-type PMC. The mutant plant *rmi2-2* did not show any defects compared with wild-type PMC in neither meiotic stages of meiosis I (pachytene to dyade) nor meiosis II (telophase II to tetrade).
(PDF)

S5 Fig Embryo analysis in At*rmi2-2 rtel1-1*. The progression of embryo development proceeds without irregularities in the double mutant *rmi2-2 rtel1-1* compared to wild-type plants.
(PDF)

S1 Table Oligonucleotides used in this study. (PDF)

S2 Table Amount of PMCs from one inflorescence. (PDF)

S1 Method Expression analysis by quantitative real-time PCR. (PDF)

S2 Method Preparation of pollen mother cells from inflorescences. (PDF)
