# Supplementary material for: The RTR Complex Partner RMI2 and the DNA Helicase RTEL1 Are Both Independently Involved in Preserving the Stability of 45S rDNA Repeats in Arabidopsis thaliana
Source: PLoS Genet. 2016 Oct 19;12(10):e1006394. doi: 10.1371/journal.pgen.1006394 (PMC5070779; doi:10.1371/journal.pgen.1006394)
Supplement: S1 Fig — (A) Gene structure of the AtRMI2 gene with three exons and two introns and a length of 884 bp. (B) The Atrmi2-1 mutant carries a multi-copy T-DNA insertion in intron II flanked on both sides by LB structures and bordered by a 2 bp duplication of chromosomal sequence. (C) The Cas9-mediated mutant Atrmi2-2 carries a 297 bp insertion in exon I, harbouring a stop codon (red box) in frame, which was validated by cDNA analysis. (PDF) [file pgen.1006394.s001.pdf]

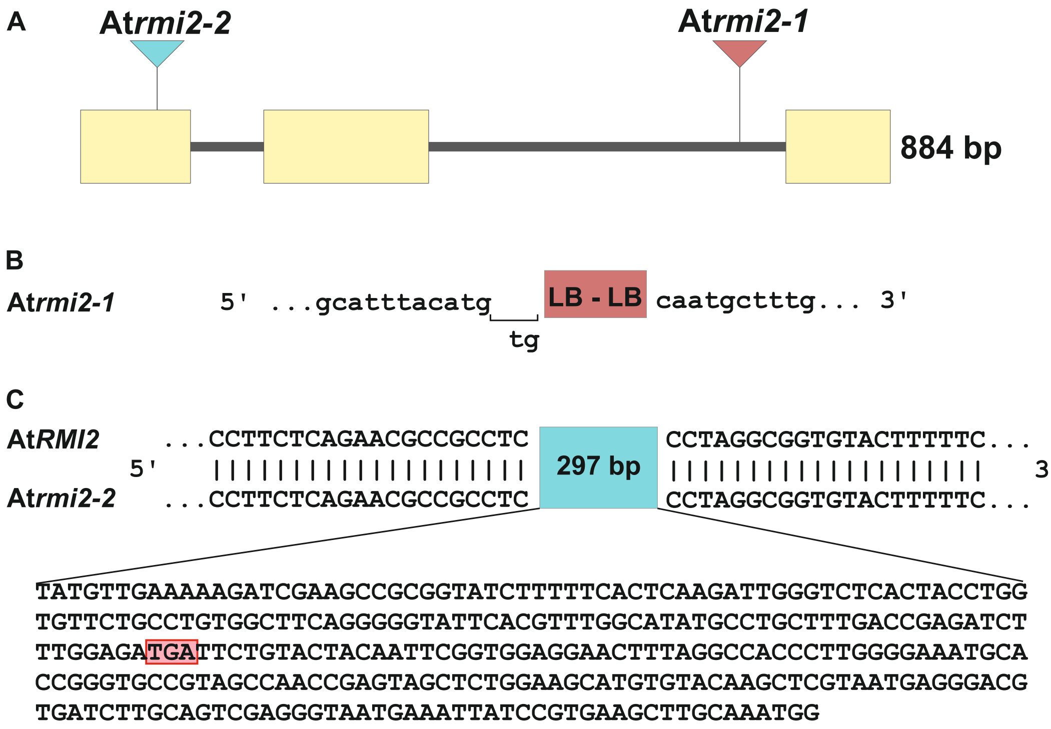
A
Atrmi2-2
Atrmi2-1
884 bp
B
Atrmi2-1
5' ...gcatttacatg
LB - LB
caatgctttg... 3'
tg
C
AtRMI2
...CCTTCTCAGAACGCCGCCTC
297 bp
CCTAGGCGGTGTACTTTTTC...
5'
3'
Atrmi2-2
...CCTTCTCAGAACGCCGCCTC
CCTAGGCGGTGTACTTTTTC...
TATGTTGAAAAAGATCGAAGCCGCGGTATCTTTTTCACTCAAGATTGGGTCTCACTACCTGG
TGTTCTGCCTGTGGCTTCAGGGGGTATTCACGTTTGGCATATGCCTGCTTTGACCGAGATCT
TTGGAGATGATTCTGTACTACAATTCGGTGGAGGAACTTTAGGCCACCCTTGGGGAAATGCA
CCGGGTGCCGTAGCCAACCGAGTAGCTCTGGAAGCATGTGTACAAGCTCGTAATGAGGGACG
TGATCTTGCAGTCGAGGGTAATGAAATTATCCGTGAAGCTTGCAAATGG
