# Supplementary figures and images for: The RTR Complex Partner RMI2 and the DNA Helicase RTEL1 Are Both Independently Involved in Preserving the Stability of 45S rDNA Repeats in Arabidopsis thaliana
Source: PLoS Genet. 2016 Oct 19;12(10):e1006394. doi: 10.1371/journal.pgen.1006394 (PMC5070779; doi:10.1371/journal.pgen.1006394)

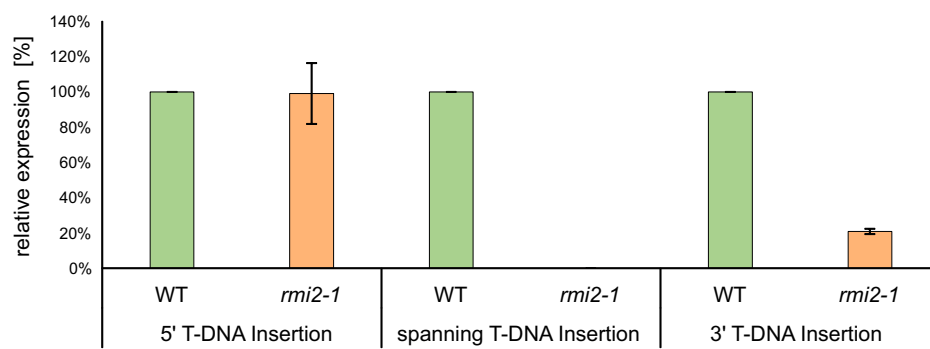

Supplement: S2 Fig — Absence of the full-length mRNA of RMI2 in the T-DNA insertion mutant rmi2-1 was tested by quantitative real-time PCR with three different primer pairs. The expression of the gene 5' from the insertion locus was comparable to wild-type expression. We could not detect a transcript spanning the T-DNA insertion. The expression 3' of the insertion locus was severely decreased. (PDF) [file pgen.1006394.s002.pdf]

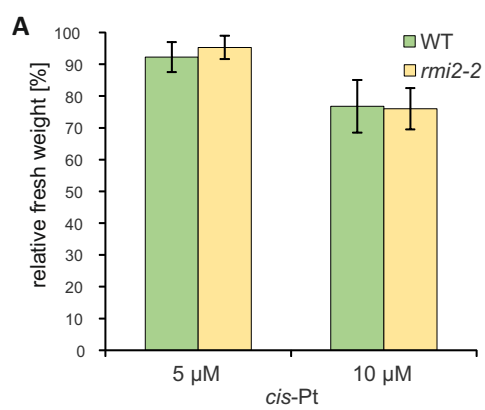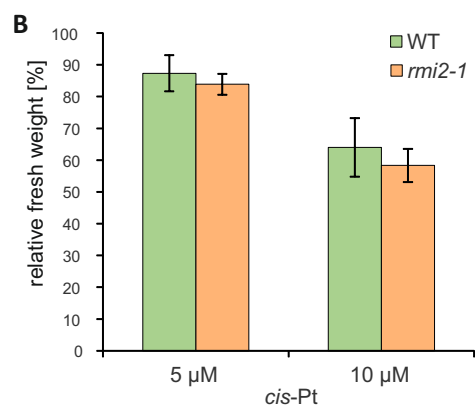

Supplement: S3 Fig — Relative fresh weight after treatment with 5 or 10 μM cis-Pt to induce intrastrand crosslink DNA-damage are depicted. Both mutant lines, the T-DNA insertion line rmi2-1 and the Cas9-induced insertion line rmi2-2 did not show elevated sensitivity compared to wild-type plants. (PDF) [file pgen.1006394.s003.pdf]

*rmi2-2*

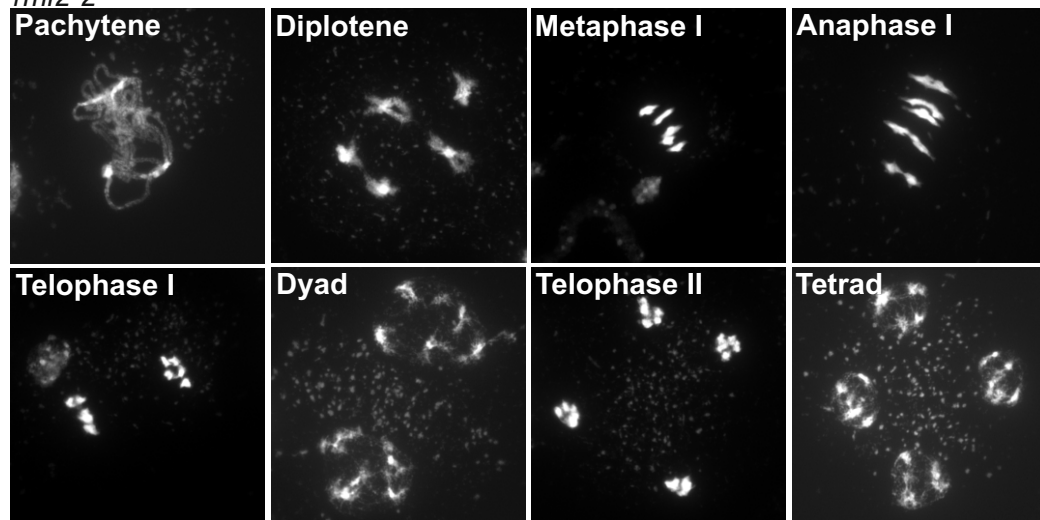

WT

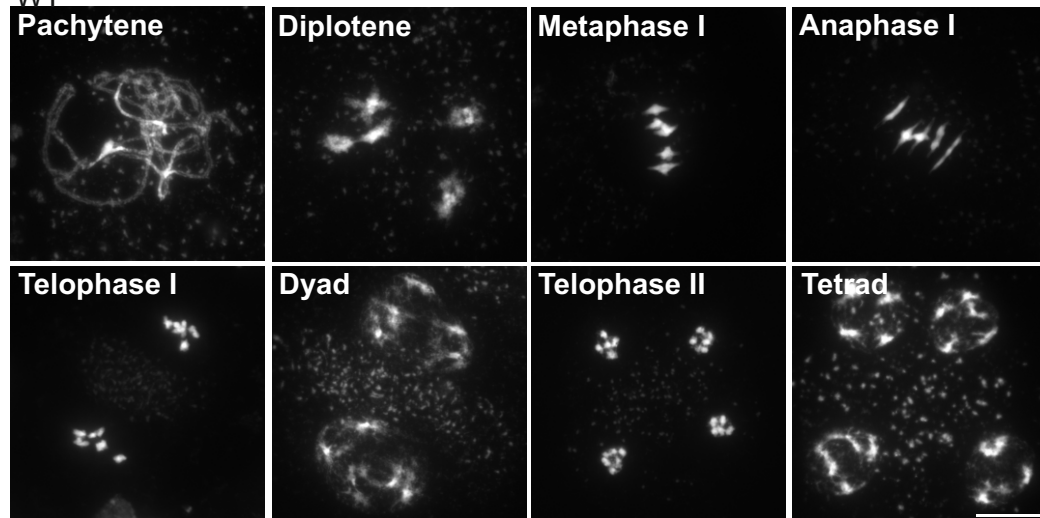

Supplement: S4 Fig — The mutant plant rmi2-2 did not show any defects compared with wild-type PMC in neither meiotic stages of meiosis I (pachytene to dyade) nor meiosis II (telophase II to tetrade). (PDF) [file pgen.1006394.s004.pdf]

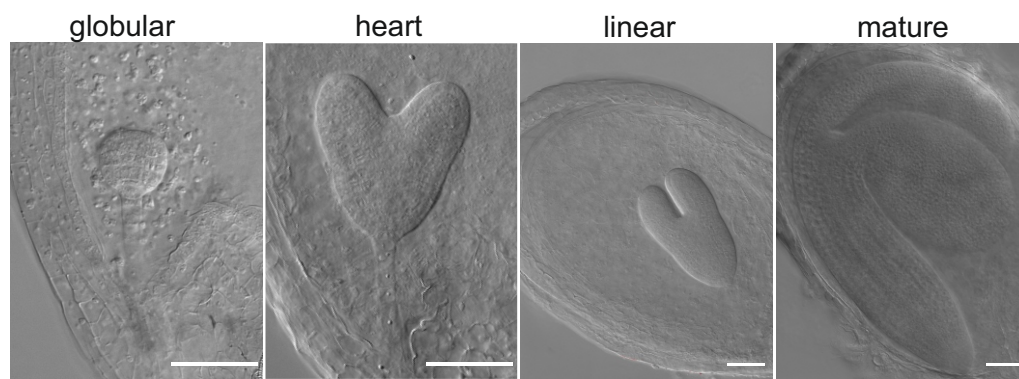

WT

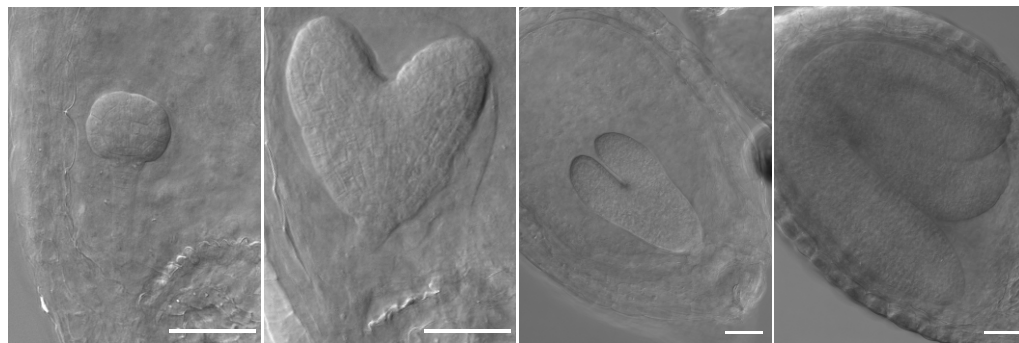

*rmi2-2 rtel1-1*

Supplement: S5 Fig — The progression of embryo development proceeds without irregularities in the double mutant rmi2-2 rtel1-1 compared to wild-type plants. (PDF) [file pgen.1006394.s005.pdf]
