# Supplementary material for: The RTR Complex Partner RMI2 and the DNA Helicase RTEL1 Are Both Independently Involved in Preserving the Stability of 45S rDNA Repeats in Arabidopsis thaliana
Source: PLoS Genet. 2016 Oct 19;12(10):e1006394. doi: 10.1371/journal.pgen.1006394 (PMC5070779; doi:10.1371/journal.pgen.1006394)
Supplement: S1 Table — (PDF) [file pgen.1006394.s006.pdf]

S1 Table. Oligonucleotides used in this study.

Target	Name	5' – 3' sequence	Analysis
Ubiquitin 10	UBQC-FW1	AACGGGAAAGACGATTAC	Analysis of 45S rDNA repeats
	UBQC-RV1	ACAAGATGAAGGGTGGAC	
**18S rDNA**	18S-FW1	CTAGAGCTAATACGTGCAACAAAC	
	18S-RV1	GAATCGAACCCTAATTCTCCG	
**5.8S rDNA**	5.8S-FW1	CGGAGTGTGGGCGGATG	
	5.8S-RV1	GTGAGGGACGACGATTTG	
**25S rDNA**	25S-FW1	GTGCGAGTCAACGGGTG	
	25S-RV1	ACCCAAGTCAGACGAACG	
RMI2	RMI2-FW1	CTAAACCGACGCCTTCTC	Genotyping At*rmi2-2*
	RMI2-RV1	GTGAGAATGTTAGATGTTAT GA	
	RMI2-FW2	CGTGTGCGAGAGATTCTTAGG	Genotyping At*rmi2-1*
	RMI2-RV2	CCCTAGGCGGTGTACTTTTTC	
	GABI-LB	TTGGACGTGAATGTAGACAC	
**5' TDNA**	5' TDNA-FW1	TTAGCGGCGGTGAAGATGC	Expression analysis *RMI2* in At*rmi2-1*
	5' TDNA-RV1	CGGTCTTTTGGAGCTCGGT	
**T-DNA spanning**	T-DNA-FW1	CTCCGTCAATGGAAATCAGG	
	T-DNA-RV1	CTTCTATGAGCCCTTGATCC	
**3' T-DNA**	3' T-DNA-FW1	TGTGGTATTTAGAAGTCATG GA	
	3' T-DNA-RV1	TCTACGGAATCAAAAGCAAG G	
