# Supplementary material for: The RTR Complex Partner RMI2 and the DNA Helicase RTEL1 Are Both Independently Involved in Preserving the Stability of 45S rDNA Repeats in Arabidopsis thaliana
Source: PLoS Genet. 2016 Oct 19;12(10):e1006394. doi: 10.1371/journal.pgen.1006394 (PMC5070779; doi:10.1371/journal.pgen.1006394)
Supplement: S2 Table — (PDF) [file pgen.1006394.s007.pdf]

S2 Table. Amount of PMCs from one inflorescence.

	Meiocytes	
**Inflorescence**	**Col-0**	***rmi2-2 rtel1-1***
1	192	3
2	33	1
3	104	3
4	86	5

	Relation	
	**Col-0**	***rmi2-2 rtel1-1***
	64	1
	33	1
	35	1
	17	1
Mean Value	37	1
p-value		0.0340
t-test		*
