# Supplementary material for: The RTR Complex Partner RMI2 and the DNA Helicase RTEL1 Are Both Independently Involved in Preserving the Stability of 45S rDNA Repeats in Arabidopsis thaliana
Source: PLoS Genet. 2016 Oct 19;12(10):e1006394. doi: 10.1371/journal.pgen.1006394 (PMC5070779; doi:10.1371/journal.pgen.1006394)
Supplement: S1 Method — (PDF) [file pgen.1006394.s008.pdf]

S1 Method. Expression analysis by quantitative real-time PCR.

RNA extraction and cDNA analysis was performed as described before. The resulting cDNA was used as template, combined with 5 µl 2x qPCR Master Mix KAPA SYBR FAST containing SYBR Green dye (Kapa Biosystems), 0.5 µl of each primer (10 mM) and distilled water in an overall volume of 10 µl. The Light Cycler 480 instrument (Roche Diagnostics) was used with a 384-well block system. Three locations in the genome were analysed by corresponding primer pairs, targeting 5' and 3' of the T-DNA insertion as well as the T-DNA insertion site itself (S1 Table). The results of two different constitutively expressed genes (At3g18780 and At4g34270) were applied for normalisation (S1 Table).
