# Supplementary material for: The RTR Complex Partner RMI2 and the DNA Helicase RTEL1 Are Both Independently Involved in Preserving the Stability of 45S rDNA Repeats in Arabidopsis thaliana
Source: PLoS Genet. 2016 Oct 19;12(10):e1006394. doi: 10.1371/journal.pgen.1006394 (PMC5070779; doi:10.1371/journal.pgen.1006394)
Supplement: S2 Method — (PDF) [file pgen.1006394.s009.pdf]

S2 Method. Preparation of pollen mother cells from inflorescences.

The preparation of PMC was performed as described in previous studies. After four weeks of cultivation in the greenhouse, primary inflorescences were harvested and preserved in fixative solution (ethanol : acetic acid, in proportion 3:1). After changing the pH of flower buds with 0.01 M citrate buffer to pH 4.5, a digestion solution was applied for 75 min at 37 °C. Three to four flower buds were transferred to a microscope slide, squashed and mixed with 7 µl of 60 % acetic acid. After heating each slide to 45 °C, fixative solution was added and microscope slide was dried. Chromatin spreads were stained with 10 µl of DAPI (4'6-Diamidin-2-phenylindol) and analysed by fluorescence microscopy.
